# Interactions of Self-Assembled *Bletilla Striata* Polysaccharide Nanoparticles with Bovine Serum Albumin and Biodistribution of Its Docetaxel-Loaded Nanoparticles

**DOI:** 10.3390/pharmaceutics11010043

**Published:** 2019-01-19

**Authors:** Guangyuan Zhang, Jin Qiao, Xin Liu, Yuran Liu, Ji Wu, Long Huang, Danyang Ji, Qingxiang Guan

**Affiliations:** Department of Pharmaceutics, School of Pharmacy, Jilin University, Changchun 130012, China; zhanggy17@mails.jlu.edu.cn (G.Z.); qiaojin17@mails.jlu.edu.cn (J.Q.); liux@jlu.edu.cn (X.L.); liuyr18@mails.jlu.edu.cn (Y.L.); wuji18@mails.jlu.edu.cn (J.W.); huanglong18@mails.jlu.edu.cn (L.H.); jidy16@mails.jlu.edu.cn (D.J.)

**Keywords:** *Bletilla striata* polysaccharide, nanoparticle, interaction, bioavailability, tissue distribution

## Abstract

Amphiphilic copolymers of stearic acid (SA)-modified *Bletilla striata* polysaccharides (BSPs-SA) with three different degrees of substitution (DSs) were synthesized. The effects of DS values on the properties of BSPs-SA nanoparticles were evaluated. Drug state, cytotoxicity, and histological studies were carried out. The affinity ability of bovine serum albumin (BSA) and the BSPs-SA nanoparticles was also characterized utilizing ultraviolet and fluorescence spectroscopy. Besides, the bioavailability and tissue distribution of docetaxel (DTX)-loaded BSPs-SA nanoparticles were also assessed. The results demonstrated that the DS increase of the hydrophobic stearic acid segment increased the negative charge, encapsulation efficiency, and drug-loading capacity while decreasing the critical aggregation concentration value as well as the release rate of docetaxel from the nanoparticles. Docetaxel was encapsulated in nanoparticles at the small molecules or had an amorphous status. The inhibitory capability of DTX-loaded BSPs-SA nanoparticles against 4T1 tumor cells was superior to that of Duopafei^®^. The ultraviolet and fluorescence results exhibited a strong binding affinity between BSPs-SA nanoparticles and bovine serum albumin, but the conformation of bovine serum albumin was not altered. Additionally, the area under the concentration–time curve (AUC_0–∞_) of DTX-loaded BSPs-SA nanoparticles was about 1.42-fold higher compared with Duopafei^®^ in tumor-bearing mice. Docetaxel levels of DTX-loaded BSPs-SA nanoparticles in some organs changed, and more docetaxel accumulated in the liver, spleen, and the tumor compared with Duopafei^®^. The experimental results provided a theoretical guidance for further applications of BSPs-SA conjugates as nanocarriers for delivering anticancer drugs.

## 1. Introduction

Polymeric self-assembled nanoparticles have been applied in drug delivery systems for several decades and have attracted massive attention in the pharmaceutical field [1,2,3,4]. Amphiphilic conjugates consisting of hydrophobic and hydrophilic segments can spontaneously self-aggregate into nanoparticles with a hydrophilic shell and a hydrophobic core due to the inter-and/or intramolecular interactions of the hydrophobic segments in aqueous media [5,6]. The hydrophobic inner domain of the self-assembly system serves as a good vehicle for poorly water-soluble drugs and prominently enhances their solubility, bioavailability, and systemic circulation time in the blood [7,8]. Besides, the hydrophilic shell can stabilize the nanoparticle drug delivery systems, avoid the clearance of nanoparticles by the reticular endothelial system, and facilitate the accumulation of drugs in tumor by enhanced permeability and retention effects [9]. Owing to these perfect properties, self-assembled nanoparticles can serve as promising chemotherapeutic carriers and exhibit great potential in improving cancer therapy [10,11].

In the recent several years, the investigations of amphiphilic synthetic copolymers have focused on their functionalities [12,13]. Meanwhile, hydrophilic polysaccharides modified with hydrophobic segments have also attracted great attention [14,15]. Both can self-aggregate into nanoparticles in an aqueous environment. However, compared with the synthetic polymers, natural polysaccharides show many beneficial biological properties, including non-toxicity, biodegradability, and non-immunogenicity [5]. In addition, polysaccharides have abundant resources in nature and are obtained easily. Specifically, most polysaccharides exhibit intrinsic specific bioadhesion [16] and perfect biocompatibility [5]. Water-soluble polysaccharides (e.g., chitosan, hyaluronic acid, and dextran) can be modified easily and display amphiphilic properties through grafting different kinds of hydrophobic moieties, which provide an alternative candidate for drug delivery systems [5].

Docetaxel is highly efficacious in the treatment of breast cancer. Duopafei^®^, a commercial docetaxel formulation using ethanol and Tween 80 as solvents, may cause some serious adverse effects, such as fluid retention, neurotoxicity, and hypersensitivity [17]. A number of promising strategies [17] including nanoparticle, nanoemulsion, liposome, and lipid-based-nanosuspensions have been extensively adopted to reduce these adverse reactions. Besides, some natural polysaccharides (dextran, hyaluronic acid, and carboxymethyl cellulose, etc.) have also been hydrophobically modified and developed as a self-assembled drug delivery system for targeting delivery docetaxel to cancer sites to overcome these deficiencies [18].

**Figure 1:**
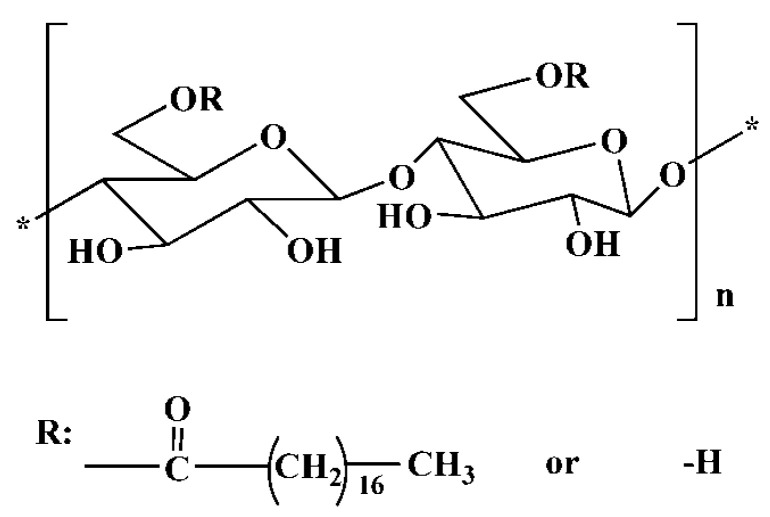
Chemical structure of stearic acid-modified *Bletilla striata* polysaccharides (BSPs-SA).

*Bletilla striata* polysaccharides (BSPs), an extract produced from the tubers of *Bletilla striata* (Thunb.) Reichb. f., striata, consist of (1,2)-α-d-mannopyranose, (1,4)-β-d-glucose, and (1,6)-β-d-galactosyl residues, and bear enormous advantages involving hydrophilicity, biodegradability, nontoxicity, and biocompatibility [19]. BSPs have been extensively used in chemical industries as gels, suspension solutions, and binders in food industries. Particularly, BSPs have been employed to target delivery drug in the pharmaceutical and biomedical field [20,21]. However, BSPs are water-soluble polymers which constrict their application as a poorly water-soluble drug carrier. To overcome this limitation and enhance the encapsulating capability of hydrophobic drugs, stearic acid (SA)-modified *Bletilla striata* polysaccharides (BSPs-SA) amphiphilic copolymers have been synthesized and characterized in our previous reports [20,21]. The structure of BSPs-SA is shown in Figure 1. Docetaxel (DTX) was chosen as the model drug to prepare DTX-loaded BSPs-SA nanoparticles. It was characterized through study of particle size and zeta potential, encapsulation efficiency and loading capacity, in vitro drug release in pH 7.4 phosphate buffer saline (PBS), in vitro cytotoxicity on Hela and HepG2 cells, and in vivo bioavailability in healthy rats [20]. The effects of the drug/carrier on the characterization of DTX-loaded BSPs-SA nanoparticles, the in vitro anticancer activity on HepG2, SW480, MCF-7, and HeLa cells, cellular uptake of DTX-fluorescein isothiocyanate (FITC) labeled-BSPs-SA nanoparticles by a flow cytometry assay, the toxicity of BSPs-SA on human umbilical vein endothelial cells, and in vitro hemolysis assay were also studied [21]. The impacts of pH values (5.0, 6.5, and 7.4) on particle size, zeta potential, in vitro release behavior, the in vitro cytotoxic effect of DTX-loaded BSPs-SA nanoparticles on A549 and MCF-7 cells, quantitative cellular uptake determined by high performance liquid chromatography, determining apoptosis, antitumor effect in vivo in mice bearing 4T1 tumor cells, and the effect of DTX-loaded BSPs-SA nanoparticles on H_2_O_2_-induced hemolysis of red blood cells were investigated [22]. The mice subjected to DTX-loaded BSPs-SA nanoparticles and Duopafei^®^ displayed an obvious reduction in tumor weight and volume in comparison to that in the model control group (*p* < 0.05), respectively. Notably, the inhibition capability of DTX-loaded BSPs-SA nanoparticles on tumor cells in vitro and antitumor effects in vivo is superior to that of Duopafei^®^ [22].

It is reported that the degree of substitution (DS) value of the hydrophobic moiety conjugated to polysaccharides has influences on the cytotoxicity, cellular uptake, and transfection efficiency of nanoparticles [23]. Besides, the morphology and particle size of nanoparticles can be affected by the DS value of hydrophobic group [24]. It is worth mentioning that particle size tends to decline along with the increment of DS value of hydrophobic segments due to enhanced hydrophobic interactions [24]. Up to now, the impacts of DS values on the physicochemical characterization of self-aggregated nanoparticles have rarely been explored [24,25].

After entering the body, nanoparticles may have certain interaction with proteins such as human serum albumin. The interaction may influence the structure and function of protein [26] as well as physicochemical characteristics of nanoparticles such as surface structure, shape, and aggregation [27]. However, the investigations on their interaction have rarely been reported [28,29,30]. Generally, bovine serum albumin is employed as a model protein due to its homologous structure with human serum albumin [31]. The interaction can be determined using fluorescence, ultraviolet spectroscopy, and isothermal titration calorimeter methods [32].

The DS value of stearic acid is defined as the number of stearic acid moieties per 100 sugar residues of BSPs, which can be calculated from the peak areas of ^1^H-Nuclear Magnetic Resonance (NMR) signals using the following equation from the literature [20]: DS (%) = (A_δ1.24_/32 + A_δ0.85_/3) / (A_δ5.43_ + A_δ4.55_) × 100%, where δ_0.85_ and δ_1.24_ ppm are separately the characteristic signals of stearic acid. A_δ1.24_ and A_δ0.85_ represent the peak area of methylene protons and methyl protons, respectively. δ_5.43_ ppm and δ_4.55_ ppm represent the special chemical shift of BSPs. A_δ4.55_ represents the peak area of hydroxyl [H (1, 4)] protons and A_δ5.43_ is the peak area of hydroxyl [H (1,6)] protons. We found that the BSPs-SA conjugates with lower DS values (<14.9%) could self-aggregate into nanoparticles. Surprisingly, BSPs-SA conjugates with higher DS values (14.9–18.5%) generated precipitates, which suggested nanoparticles could not be achieved. Consequently, it was vitally essential to acquire amphiphilic conjugates supplying the desired DS value of the hydrophobic segments to obtain perfect nanoparticles. We have successfully prepared three kinds of BSPs-SA conjugates with DS values of 4.98%, 9.18%, and 12.94%, expressed as BSPs-SA_4.98_, BSPs-SA_9.18_, and BSPs-SA_12.94_, respectively.

Herein, the aim of the present study is to investigate the effects of DS values of stearic acid segment on the physicochemical characteristics including critical aggregation concentration, particle size, zeta potential, encapsulation efficiency, drug-loading capacity, and drug release behaviors of self-assembled BSPs-SA nanoparticles. Moreover, the status of docetaxel in DTX-loaded BSPs-SA nanoparticles and their cytotoxicity were evaluated. Besides, bovine serum albumin was selected as a model protein to determine the binding ability with BSPs-SA nanoparticles. Furthermore, pharmacokinetics and tissue distribution of DTX-loaded BSPs-SA nanoparticles in mice bearing the 4T1 tumor were compared with Duopafei^®^.

## 2. Materials and Methods

### 2.1. Materials

Acetonitrile was purchased from Thermo Fisher Scientific Co., Ltd (Rockford, IL, USA). Docetaxel was supplied by Shanghai Boylechem Co., Ltd (Shanghai, China, a purity of 99.6%). Duopafei^®^ was bought by Qilu Pharmaceutical Co., Ltd. (1 mL:20 mg, Jinan, China). *Bletilla striata* polysaccharides were provided by Shanxi Pioneer Biotech Co., Ltd (Xi’an, China, a purity of 98.5%). Dulbecco’s Modified Eagle Medium, fetal bovine serum, trypsin, and phosphate buffer saline were all purchased from Thermo Fisher Scientific Co., Ltd (South Logan, UT, USA). Dimethyl sulfoxide, ethanol, and methanol were provided by Tianjin Guangfu Fine Chemical Research Institute (Tianjin, China). 3-(4,5-dimethyl-2-thiazolyl)-2,5-diphenyl-2H-tetrazolium bromide was bought from Energy Chemical Co., Ltd (Shanghai, China). The lactate dehydrogenase assay kit was provided by Nanjing Jiancheng Bioengineering Institute (Nanjing, China). The reactive oxygen species assay kit was supplied by Beyotime biotechnology (Nanjing, China).

Six-to-eight-week-old BALB/c female mice weighing 18–22 g (Changchun Institute of Biological Products Co., Ltd., Changchun, China) were used in the study. All mice were fed at 70 ± 5% relative humidity and 25 ± 2 °C under natural alternative day and night conditions for one week before experiments. The experiments were conducted in full compliance with the principles of the Institutional Animal Care and Use Committee of Jilin University (license No. SCXK-(JI) 2017-0012, 12 April 2017). All efforts were taken to minimize suffering.

### 2.2. Preparation of DTX-Loaded BSPs-SA Nanoparticles

The BSPs-SA nanoparticles solution was acquired by dialysis method as we reported previously [21]. In brief, BSPs-SA conjugates were dissolved with dimethyl sulfoxide solution and then put in dialysis bags for dialyzing procedure via deionized water. The resultant BSPs-SA nanoparticles solution was filtered through a 0.45-μm membrane filter and adjusted to 0.5 mg/mL of concentration with deionized water. Docetaxel dissolved with chloroform/absolute ethanol (3:1, *v*/*v*) was added into BSPs-SA nanoparticles solution at a drug and carrier mass ratio of 1:6. The BSPs-SA nanoparticles solution was magnetically stirred for 24 h. DTX-loaded BSPs-SA nanoparticles were harvested after the solvent was evaporated.

### 2.3. Characterization of Nanoparticles

#### 2.3.1. Particle Size and Zeta Potential

The hydrodynamic diameter and zeta potential of DTX-loaded BSPs-SA nanoparticles at the concentration of 0.5 mg/mL were assayed on a dynamic light scattering particle size analyzer (Zetasizer Nano ZS, Malvern Instruments, Malvern, UK) at 633 nm at 25 °C. Experiments were performed at least in triplicate and expressed as the mean +/− the standard deviation (SD).

#### 2.3.2. Morphology and Critical Aggregation Concentration

The morphology of nanoparticles was observed on a JEM-2010 (JEOL, Tokyo, Japan) transmission electron microscope at 80 kV voltage. The samples were prepared by dropping 10 μL nanoparticles solution (0.5 mg/mL) on a copper grid, which were air-dried and stained with 1% phosphotungstic acid for a 10-min duration. Samples were measured after the excess moisture was removed with a filter paper. Critical aggregation concentration values were detected by fluorescence spectroscopy [20].

#### 2.3.3. Determination of Encapsulation Efficiency and Drug-Loading Capacity

Docetaxel was extracted from 1.0 mL of DTX-loaded BSPs-SA nanoparticles at the concentration of 0.5 mg/mL using two-fold volumetric ethanol through vortex for 2 min and then underwent 5 min ultrasound. The mixture solution was centrifuged at 10,012.5× *g* for 10 min. The supernatant was collected for detecting the total weight of docetaxel (DTX*_t_*). Meanwhile, DTX-loaded BSPs-SA nanoparticles (1.0 mL) were centrifuged at 10,012.5× *g* for 10 min, and the supernatant was collected for measuring free docetaxel (DTX*_f_*). Docetaxel content was determined by high-performance liquid chromatography equipped with a SPD-20A UV detector and LC-20AT pump controlled through Lab-solution software (Shimadzu, Tokyo, Japan). A Diamonsil C_18_ column (5 μm, 4.6 mm × 250 mm, Dikma, Beijing, China) was maintained at 30 °C. The mobile phase consisted of deionized water and acetonitrile at a volumetric ratio of 40:60. The wavelength of measurement was set at 230 nm with a flow rate of 1.0 mL/min. All experiments were performed in triplicate. The percentages of encapsulation efficiency and drug loading capacity were calculated with the following equations [20]:

Encapsulation Efficiency (%) = (DTX*_t_* − DTX*_f_*)/DTX*_t_* × 100% (1)

Drug-Loading Capacity (%) = (DTX*_t_* − DTX*_f_*)/weight of copolymer nanoparticle × 100% (2)

where DTX*_t_* (mg) is the total weight of docetaxel and DTX*_f_* (mg) is the unentrapped docetaxel in the supernatant.

### 2.4. Determination of Drug Release in Vitro

The release of docetaxel from DTX-loaded BSPs-SA nanoparticles with three DS values of stearic acid moiety was conducted using dialysis bag method as reported in our previous publication [20]. Briefly, 3 mL of DTX-loaded BSPs-SA nanoparticles and Duopafei^®^ with an equivalent concentration of 100 μg/mL docetaxel were placed inside dialysis bags (*M*_W_= 8–12 kDa), which were immersed in beakers filling with 100 mL pH 7.4 phosphate-buffered saline containing 0.2% Tween 80. Afterwards, these beakers were horizontally shaken in a 37 ± 0.5 °C water bath at a shaking speed of 100 rpm. At predetermined time points, the release medium (5 mL) was removed and immediately replaced with an equal fresh medium at 37 ± 0.5 °C. The amount of docetaxel in the filtrate was analyzed by high-performance liquid chromatography as described in Section 2.3.3. However, the mobile phase of isometric elution was composed of deionized water and acetonitrile at the volumetric ratio of 50:50 to avoid the interference of Tween 80 in release medium. Cumulative release percentage (*Q*) was calculated with the Equation (3) and the release profiles were drawn. Sink conditions remained during the experimental duration.

(3) $$Q=\left[V_{0}C_{i}+V\sum C_{\left(i-1\right)}\right]/m\times 100\%$$

where *m* represents the total amount of docetaxel in DTX-loaded BSPs-SA nanoparticles or Duopafei^®^ inside the dialysis bags, V_0_ represents the total volume of release media, C_i_ represents drug concentration in release medium at time *i*, *Q* represents the cumulative release percentage of docetaxel, and V represents the volume of sample per time, respectively.

### 2.5. X-Ray Diffraction and Differential Scanning Calorimetric Analysis

The states of docetaxel in nanoparticles were monitored by X-ray diffraction and differential scanning calorimetric analysis. Measurements were carried out using a Bruker D8 advance X-ray diffractometer (Bruker, Karlsruhe, German) providing with a graphite monochromatized Cu Kα radiation (λ = 1.54 nm). All samples were scanned in the range of 4–60° (2 θ) at a scanning speed of 4 °C/min. The X-ray tube was operated at a 50-kV voltage and a 200-mA current.

Differential scanning calorimetric analysis was recorded on a NETZSCH instrument (STA 449F3, Ahlden, German) with a heating rate of 10 °C/min in the range of 40–350 °C.

### 2.6. Cell Viability

The 3-(4,5-dimethyl-2-thiazolyl)-2,5-diphenyl-2H-tetrazolium bromide (MTT) method was used to assay the cytotoxicity of DTX-loaded BSPs-SA nanoparticles and Duopafei^®^ on 4T1 cells. 4T1 cells were seeded at a density of 5 × 10^4^ viable cells/well in a 96-well plate and cultured for 24 h at 37 °C with 5% CO_2_ before further treatments. The cells were incubated with DTX-loaded BSPs-SA nanoparticles, Duopafei^®^, and blank BSPs-SA nanoparticles for 72 h, respectively. To measure cell viability, 20 μL of 5 mg/mL MTT phosphate buffer saline was placed into each well and continuously incubated for 4 h. The media were gently removed and 150 μL of dimethyl sulfoxide were added to dissolve formazan crystals. The optical density (OD) of each well was determined utilizing a Bio-Tek microplate reader (Bio-Tek FL600, Bio-Tek, Winooski, VT, USA) at 492 nm. Cell viability (%) was calculated by the following Equation (4):

(4) $$Cell\;viability\left(\%\right)=\left(OD_{492,\;sample}-OD_{492,\;blank}\right)/\left(OD_{492,\;control}-OD_{492,\;blank}\right)\times 100\%$$

where OD_492, sample_ is the OD value of Duopafei^®^, blank BSPs-SA nanoparticles, or DTX-loaded BSPs-SA nanoparticles; OD_492, control_ is the OD value of untreated cells; OD_492, blank_ is the OD value of Dulbecco’s Modified Eagle Medium.

### 2.7. Assay of Lactate Dehydrogenase

The 4T1 cells were plated at a density of 1 × 10^5^ viable cells/well in a 12-well plate and cultured for 24 h at 37 °C with 5% CO_2_ prior to experiments. The cells were then treated with blank BSPs-SA nanoparticles, Duopafei^®^, or DTX-loaded BSPs-SA nanoparticles at the docetaxel dose of 2 μg/mL. The incubated media were collected at predetermined time points for the determination of lactate dehydrogenase. The lactate dehydrogenase contents of the extracellular incubation media were assayed by lactate dehydrogenase assay kit as described in manufacturer’s instructions [33]. The absorbance values of lactate dehydrogenase were detected by a microplate reader (Bio-Tek FL600, Bio-Tek, Winooski, VT, USA) at 450 nm.

### 2.8. Reactive Oxygen Species

The intracellular reactive oxygen species generation was determined by the 2’,7’-dichlorofluorescein-diacetate (DCFH-DA) method [34]. The 4T1 cells were treated with the same conditions as in Section 2.7 described above. The 4T1 cells were digested by 0.25 % trypsin for 3 min at 37 °C and the reaction was terminated by the addition of Dulbecco’s Modified Eagle Media containing 10% fetal bovine serum. All cells were then flushed with phosphate buffer saline three times and centrifuged at 628.75× *g* for 3 min. Then the supernatants were discarded and cells underwent continual incubation with 1 mL of 10 μmol/L DCFH-DA solution for 30 min at 37 °C in dark. After the DCFH-DA solution was removed, the cells were washed using cold phosphate buffer saline three times and maintained in 1 mL of Dulbecco’s Modified Eagle Media for determination. The fluorescence analysis was performed by a fluorescence spectrophotometer (Shimadzu RF-5301, Shimadzu, Kyoto, Japan). The excitation wavelength was set at 490 nm, and the emission wavelength was 530 nm. The slit widths for emission and excitation were both 5.0 nm.

### 2.9. Histological Examination

In total, 20 mice were randomly divided into a control group (*n* = 10) and a treatment group (*n* = 10). The treatment group received 200 mg/kg BSPs-SA conjugates (*n* = 10) via tail vein injection and potential organ toxicity was evaluated. The mice were sacrificed 15 days post administration. The heart, liver, spleen, lung, and kidney were immediately harvested, washed with saline solution, and immersed in 4% paraformaldehyde in phosphate buffer saline for 24 h at 4 °C. The fixed organs were embedded in paraffin, cut to 10 mm, and stained with hematoxylin and eosin for 2 min and 10 s on glass slides to assess histological differences between the treated group and normal control group.

### 2.10. Fluorescence and Ultraviolet (UV) Measurement

Bovine serum albumin solution at the concentration of 0.33 mg/mL and BSPs-SA nanoparticles solution at 0.1–0.7 mg/mL concentrations were prepared using deionized water, respectively. Afterwards, bovine serum albumin solution was added into BSPs-SA nanoparticles solution and horizontally shaken for 6 h incubation at 37 °C with a shaking speed of 100 rpm. The UV spectra were recorded on a UV spectrophotometer (Shimadzu 1200, Shimadzu, Kyoto, Japan) in the wavelength range of 230–298 nm. The fluorescence results were monitored on a fluorescence spectrophotometer (Shimadzu RF-5301, Shimadzu, Kyoto, Japan) utilizing deionized water as the blank probe. The excitation wavelength was set at 293 nm, and the emission spectra were recorded in the range of 300–450 nm at an integration time of 1.0 s. The slit widths of emission and excitation were both 3.0 nm.

### 2.11. Pharmacokinetics and Tissue Distribution

BALB/c female mice bearing 4T1 cancers were chosen to evaluate docetaxel distribution in tissues. All mice were subcutaneously injected at the right axillary site with 0.1 mL of tumor cell suspension (5 × 10^6^ cells/ mL). The tumor volume was calculated using the equation: *V* = (*w* × *a* × *b*)/2, where ‘*w*’ is the height of the tumor, ‘*a*’ is at the widest point and ‘*b*’ the smallest dimension. When the tumor volumes were approximately 100–200 mm^3^, mice (*n* = 5) were administered 25 mg/kg Duopafei^®^ or 25 mg/kg DTX-loaded BSPs-SA nanoparticles via the tail vein. At 10, 30, 60, 90, 120, 180, 240, and 360 min post injection, the mice were anesthetized by diethyl ether inhalation and blood samples were collected from the retro-orbital plexus. Blood samples were immediately centrifuged for 15 min at 851.75× *g* and plasma was harvested and stored at −20 °C until analysis. All mice were then euthanized by cervical dislocation and the heart, liver, kidney, lung, spleen, and tumor were harvested, cleaned, weighed, and homogenized (PB100 homogenizer, Prima, London, UK) in 1 mL of a 0.9% NaCl solution. All samples were stored at −20 °C until analysis.

### 2.12. Serum and Tissue Sample Analysis

The standard solution was achieved by dissolving docetaxel in methanol at the concentration of 1 mg/mL. Subsequently, appropriate volumes of standard solution were added in the blank plasma specimen to prepare a series of concentrations for docetaxel in the range of 0.1–50 μg/mL. The limits of detection and quantification were defined as the signal to noise (S/N) ratio of 3 and 10, respectively. Briefly, docetaxel in plasma specimen was extracted by adding 1.5-fold volumetric methanol and followed a vortex-mixing of 3 min. The mixed solution had undergone a 10,012.5× *g* centrifugation for 10 min and the harvested supernatant was concentrated under nitrogen. The residue was dissolved with 200 μL methanol and then centrifugated for monitoring docetaxel through high-performance liquid chromatography as described in Section 2.3.3. However, the mobile phase was composed of deionized water and acetonitrile at the volumetric ratio of 56:44 to acquire desired resolution between the peak of docetaxel and the other peaks conformed to the requirements.

Tissue samples were handled as the following procedure. Briefly, 1.0 mL of 0.9% NaCl was separately added into each harvested tissue sample following homogenization treatment. The resultant tissue samples were extracted with 10-fold volumetric methanol and underwent vortex-mixing for 3 min. The further procedure was similar with that of the plasma specimen as described.

### 2.13. Statistical Analysis

All pharmacokinetic parameters were analyzed using DAS 2.1 pharmacokinetic software (Chinese Pharmacological Society, Nanjing, China). Tissue–plasma concentration ratios (*K*p) were calculated to evaluate targeted tissue distribution. Experiments were performed at least in triplicate and data are represented as the mean +/− standard deviation. Statistical significance was analyzed by the Student’s *t*-test.

## 3. Results and Discussion

### 3.1. Characterization of Nanoparticles

**Figure 2:**
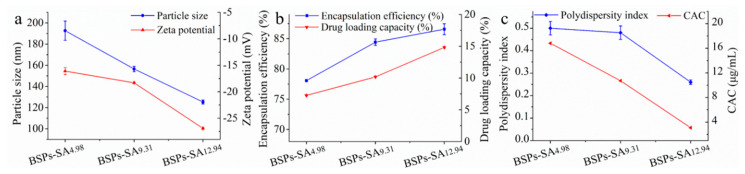
The effects of the degree of substitution (DS) values of stearic acid moieties on particle size and zeta potential (**a**), encapsulation efficiency and drug-loading capacity (**b**), and polydispersity index values and critical aggregation concentration (CAC) values (**c**) of BSPs-SA nanoparticles. Data are shown as mean ± SD. (*n* = 3).

The effects of DS on average particle size and zeta potential are presented in Figure 2a. DTX-loaded BSPs-SA nanoparticles with the DS values of 4.98%, 9.31%, and 12.94% exhibited average sizes of 192.70 ± 9.08 nm, 156.50 ± 2.52 nm, and 125.29 ± 1.89 nm, corresponding to the polydispersity index values of 0.50 ± 0.03, 0.48 ± 0.03, and 0.26 ± 0.01, respectively. The results demonstrated that the particle sizes and polydispersity index values of DTX-loaded BSPs-SA nanoparticles decreased as the DS values of stearic acid increased. This may be the result of a tighter interaction between the hydrophobic stearic acid domains of the nanoparticle due to the increase of DS values, indicating the formation of more intensive hydrophobic cores [25]. The DTX-loaded BSPs-SA nanoparticles carried negative charges. The zeta potentials of DTX-loaded BSPs-SA nanoparticles with the DS values of 4.98%, 9.31%, and 12.94% were −16.10 ± 0.66 mV, −18.30 ± 0.10 mV, and −26.92 ± 0.18 mV, respectively. Drug nanoparticles carried more negative charges and the absolute values of zeta potentials were higher accompanying the increase of DS values. There are three probable reasons. One possibility is that BSPs carry a negative charge of −12.6 ± 1.99 mV. Another reason is possibly ascribed to isoelectric points. The isoelectric points of BSPs and BSPs-SA are 3.0 and 4.0, respectively. This may result in BSPs-SA and DTX-loaded BSPs-SA to possess a more negative charge. The third reason is that stearic acid carries many negative charges with a zeta potential of -5.43 ± 0.45 mV.

The effects of DS values on the encapsulation efficiency and drug loading capacity are shown in Figure 2b. Drug-loading capacity and encapsulation efficiency increased from 7.25 ± 0.08% to 14.82 ± 0.13% and from 78.07 ± 0.10% to 86.57 ± 0.90%, respectively, with a DS range of 4.98–12.94%, which may be attributed to more hydrophobic substitution resulting in stronger hydrophobic cores and easier self-assembly into nanoparticles [35]. As shown in Figure 2c, the critical aggregation concentration values of the BSPs-SA conjugates decreased from 16.81 μg/mL to 3.09 μg/mL with an increase in DS with hydrophobic stearic acid from 4.98% to 12.94%, which could be due to the formation of higher hydrophobic interaction.

**Figure 3:**
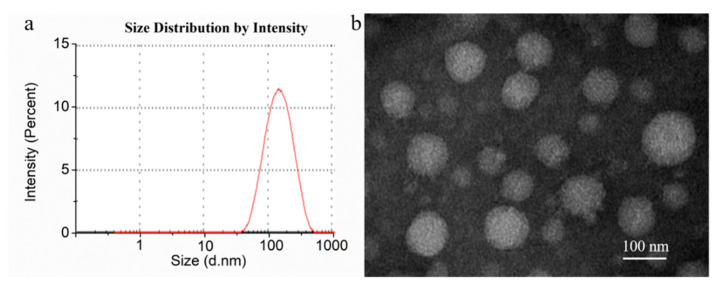
Size distribution (**a**) and transmission electron microscope image (**b**) of docetaxel (DTX)-loaded BSPs-SA_12.94_ nanoparticles. The transmission electron microscope image is shown with a magnitude 80,000× and scale of 100 nm.

The hydrodynamic particle diameter of DTX-loaded BSPs-SA_12.94_ nanoparticles was about 125.29 ± 1.89 nm, which exhibited a unimodal size distribution (Figure 3a). DTX-loaded BSPs-SA nanoparticles were spherical shapes as shown in Figure 3b. Notably, the mean particle diameter measured by dynamic light scattering appeared to be a bit larger than that measured by transmission electron microscope, which is likely due to the drying procedure during the procedure of sample preparation [25].

### 3.2. Drug Release in Vitro

**Figure 4:**
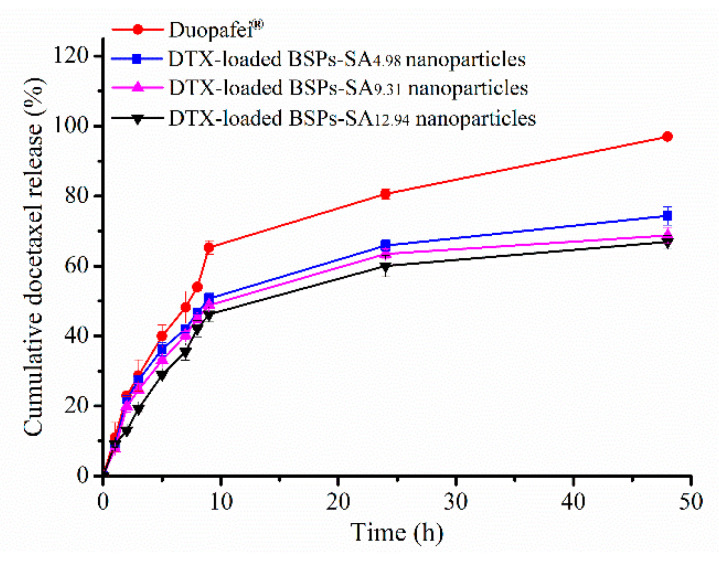
In vitro release profiles of docetaxel from Duopafei^®^ and DTX-loaded BSPs-SA nanoparticles with different DS values of stearic acid in pH 7.4 phosphate-buffered saline containing 0.2% of Tween 80 at 37 °C ± 0.5 °C. Results are expressed as mean ± SD. (*n* = 3).

The release profiles of docetaxel from Duopafei^®^ and DTX-loaded BSPs-SA nanoparticles with different DS values are compared and presented in Figure 4. The docetaxel release rate of Duopafei^®^ from the dialysis bag was greatly faster than that of DTX-loaded BSPs-SA nanoparticles at 24 h (80% vs. 60%). The docetaxel amount released from the nanoparticles followed an initial rapid release and then a slower constant release, which is beneficial for clinical application. The release pattern is similar with the results for pH 5.0, 6.0, and 7.4 phosphate-buffered saline in our previous publication [22]. We have proved that DTX-loaded BSPs-SA nanoparticles had a pH-sensitive release. Namely, the release rate of docetaxel from DTX-loaded BSPs-SA nanoparticles in vitro is accelerated in acidic media compared with that in pH 7.4 phosphate buffer solution (physiological media). Nevertheless, no remarkable differences were observed in the release percentages of Duopafei^®^ among the three corresponding release media [22]. The differences of docetaxel release rates between Duopafei^®^ and DTX-loaded BSPs-SA nanoparticles were mainly due to the core–shell structure of BSPs-SA nanoparticles. Lipophilic docetaxel was encapsulated into the hydrophobic core of BSPs-SA nanoparticles, and the drug was released slowly due to diffusion and dissolution [36]. The rapid release during the initial 9-h release from the DTX-loaded BSPs-SA nanoparticles might be attributable to the non-encapsulated drug and diffusion close to the surface of nanoparticles and represented a drug release of approximately 50%. The cumulative release percentages of docetaxel from DTX-loaded BSPs-SA_4.98_, DTX-loaded BSPs-SA_9.31_, and DTX-loaded BSPs-SA_12.94_ nanoparticles were 74.34%, 68.77%, and 66.93% at 48 h, respectively. The release percentage corresponded with the DS value, showing a slight decrease with an increase in DS value. Again, this is likely due to the increased amount of stearic acid in copolymers resulting in a more hydrophobic core and tighter interaction between hydrophobic stearic acid and docetaxel [24]. The phenomenon might be related to coulombic interactions including hydrogen bonding and hydrophobic interactions that bound the drug to the nanoparticles [37]. Considering particle size, drug-loading capacity, encapsulation efficiency, and the cumulative release percentage of nanoparticles with different DS values, BSPs-SA_12.94_ conjugates were chosen for the further experiments.

### 3.3. X-Ray Diffraction Analysis

**Figure 5:**
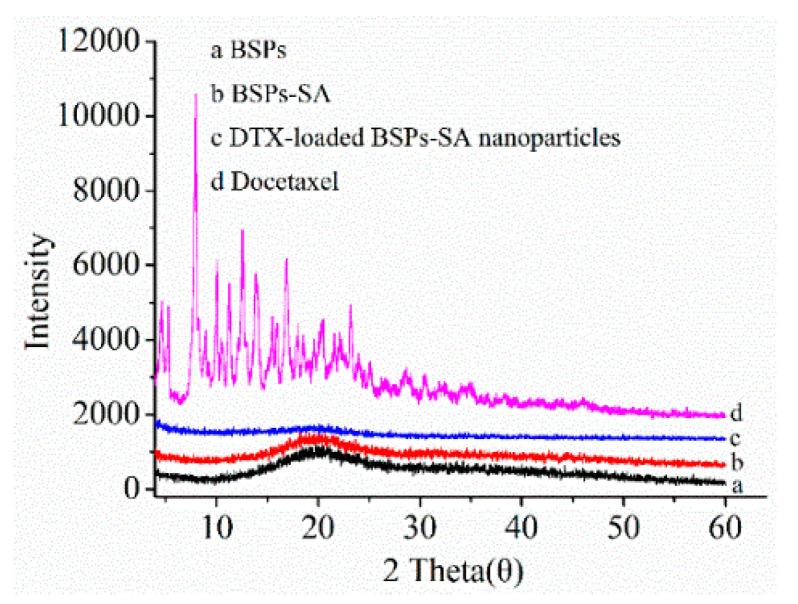
X-ray diffraction spectra (a: BSPs, b: BSPs-SA, c: DTX-loaded BSPs-SA nanoparticles, d: docetaxel).

To analyze the state of docetaxel in the BSPs-SA nanoparticles, X-ray diffraction analyses were carried out for BSPs, BSPs-SA, DTX-loaded BSPs-SA nanoparticles, and docetaxel, respectively. In Figure 5, BSPs showed a broad peak at 21.12°. BSPs-SA exhibited a broad peak of 19.18°. The peak difference between BSPs and BSPs-SA implied the formation of a new substance. Docetaxel displayed nine typical crystal peaks of 4.66°, 5.38°, 8.02°, 10.12°, 11.28°, 12.56°, 14.06°, 16.96°, and 23.18° and numerous small peaks between 24° and 40°. All typical crystal peaks of docetaxel in the lyophilized DTX-loaded BSPs-SA nanoparticles disappeared and only left a single diffraction peak, which were similar to those of the lyophilized blank BSPs-SA nanoparticles. The results suggested that docetaxel was dispersed as small molecules or in an amorphous state in the nanoparticles.

### 3.4. Differential Scanning Calorimetric Analysis

**Figure 6:**
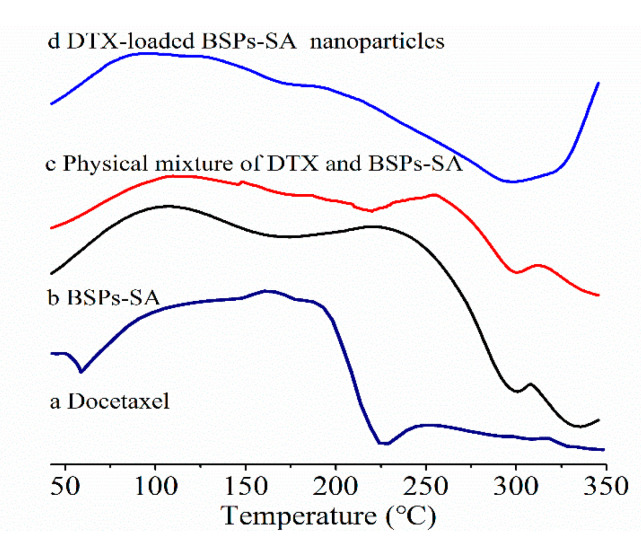
Differential scanning calorimetric curves (a: docetaxel, b: BSPs-SA, c: physical mixture of DTX and BSPs-SA, d: DTX-loaded BSPs-SA nanoparticles).

The results of differential scanning calorimetric analysis for docetaxel, BSPs-SA, the physical mixture of docetaxel and BSPs-SA, and DTX-loaded BSPs-SA nanoparticles are presented in Figure 6. Docetaxel displayed two endothermic peaks at 60 °C and 225 °C, indicating the crystalline nature of the drugs. The endothermic melting peaks of BSPs-SA appeared at 175 °C and 300 °C, respectively. For the physical mixture of docetaxel and BSPs-SA, we observed a similar endothermic peak at 225 °C corresponding to docetaxel. Nevertheless, the endothermic peak at 225 °C disappeared in the thermograms of DTX-loaded BSPs-SA nanoparticles. This phenomenon revealed that docetaxel might exist in an amorphous or disordered crystalline state after being entrapped in BSPs-SA nanoparticles.

### 3.5. Cell Viability

**Figure 7:**
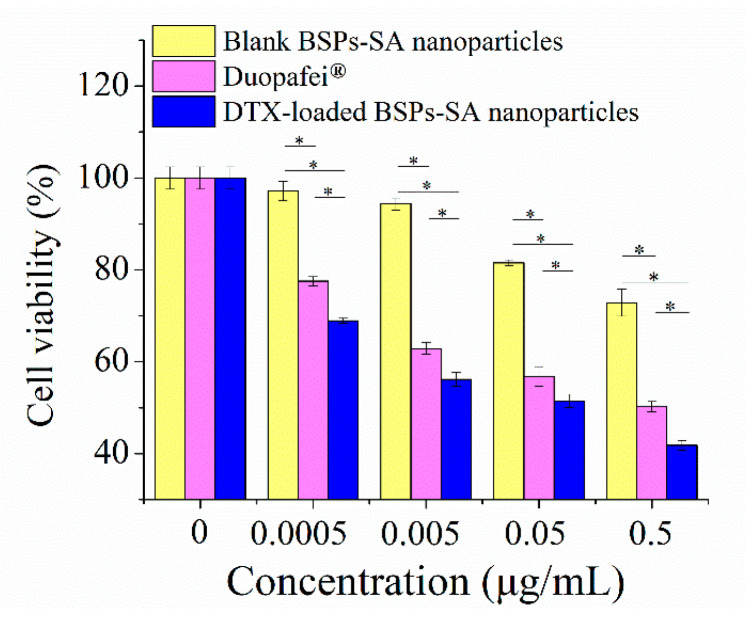
Inhibitory effects of blank BSPs-SA nanoparticles, Duopafei^®^, and DTX-loaded BSPs-SA nanoparticles on the cell viability of 4T1 tumor cells. The 4T1 cells were cultured in 96-well plate and incubated with different doses of blank BSPs-SA nanoparticles, Duopafei^®^ and DTX-loaded BSPs-SA nanoparticles for 48 h, followed by the MTT assay. Results were expressed as mean ± SD. (*n* = 6, * *p* < 0.05).

In this study, we determined the effects of Duopafei^®^ and DTX-loaded BSPs-SA nanoparticles with equivalent docetaxel concentrations of 0.0005–0.5 μg/mL against 4T1 cells through MTT assay. The results are shown in Figure 7. Both Duopafei^®^ and DTX-loaded BSPs-SA nanoparticles decreased 4T1 cell viability in a dose-dependent manner. However, the DTX-loaded BSPs-SA nanoparticles were significantly more potent than Duopafei^®^ (* *p* < 0.05). One possible reason is that the DTX-loaded BSPs-SA nanoparticles are internalized into cells via receptor mediated endocytosis [38] resulting in enhanced intracellular accumulation of the drug into tumor cells [7,39]. Another possibility is that the sustained release of docetaxel from DTX-loaded BSPs-SA nanoparticles leads to a higher efficacy. Interestingly, we found that blank BSPs-SA nanoparticles exhibited a modest inhibitory effect on cancer cell viability, which may be dominantly related to the anticancer activity of BSPs in itself [40]. Importantly, BSPs-SA conjugates appeared better biocompatible in our previous studies [21].

### 3.6. Assay of Lactate Dehydrogenase

**Figure 8:**
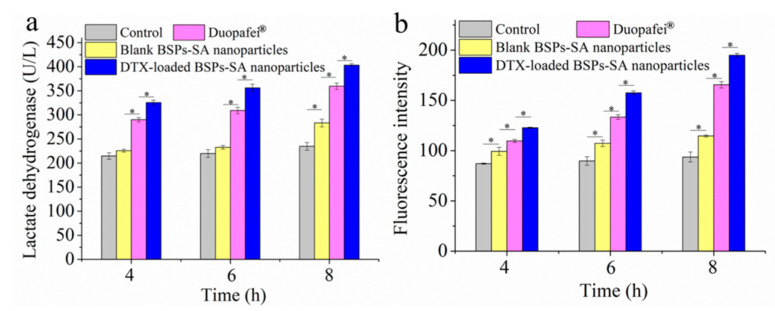
Lactate dehydrogenase release assays (**a**) and the mean fluorescence intensity of reactive oxygen species generation using 2’,7’-dichlorofluorescein-diacetate (DCFH-DA) staining (**b**) on 4T1 cells incubated with Dulbecco’s Modified Eagle Media (control), blank BSPs-SA nanoparticles, Duopafei^®^, and DTX-loaded BSPs-SA nanoparticles after 4 h, 6 h, and 8 h, respectively. The experiments were repeated in triple, results are shown as mean values ± SD. (*n* = 6, * *p* < 0.05).

Lactate dehydrogenase is an intracellular enzyme, which can be used as an important indicator of membrane integrity [41]. When cell death occurs, large amounts of intracellular lactate dehydrogenase may be released into the media [42]. As shown in Figure 8a, lactate dehydrogenase concentrations in the incubation media of 4T1 cells exposed to Duopafei^®^ and DTX-loaded BSPs-SA nanoparticles (359.71 ± 6.58 U/L and 403.5 ± 3.03 U/L) were 1.53- and 1.71-fold higher compared with those of the control group (234.74 ± 8.13 U/L) at 8 h, respectively. It was found that the tumor cells treated with Duopafei^®^ or DTX-loaded BSPs-SA nanoparticles could remarkably reduce the viability of 4T1 tumor cells and enhance the levels of extracellular lactate dehydrogenase. It is likely that the DTX-loaded BSPs-SA nanoparticles can be uptaken due to enhanced permeability and retention effects [9], resulting in an increased DTX intracellular accumulation. Another reason is that docetaxel sustained release in DTX-loaded BSPs-SA nanoparticles gives rise to greater efficacy in vitro.

### 3.7. Measurement of Reactive Oxygen Species

Reactive oxygen species, an important characteristic of intrinsic apoptosis, can induce cell death [43]. Figure 8b exhibits the levels of reactive oxygen species production after incubation with blank BSPs-SA nanoparticles, Duopafei^®^, and DTX-loaded BSPs-SA nanoparticles, respectively. Reactive oxygen species generation levels were expressed as the fluorescence intensity of dichlorofluorescein, which was the oxidized product of DCFH-DA. After exposure to Duopafei^®^ or DTX-loaded BSPs-SA nanoparticles, the generation of reactive oxygen species evidently manifested a time-dependent increase. The 4T1 tumor cells exposed to blank BSPs-SA nanoparticles, Duopafei^®^, and DTX-loaded BSPs-SA nanoparticles exhibited a significant enhancement in reactive oxygen species production with a 1.22-, 1.77-, and 2.08-fold increase, respectively, in dichlorofluorescein fluorescence in comparison to the cells treated with Dulbecco’s Modified Eagle Media alone at 8 h. Reactive oxygen species contents in DTX-loaded BSPs-SA nanoparticles group were higher than that of Duopafei^®^ group (*p < 0.05) at 4 h, 6 h, and 8 h. The results hinted that the DTX-loaded BSPs-SA nanoparticles induced greater oxidative damage to 4T1 cells than that of Duopafei^®^, which might further induce more apoptosis of 4T1 tumor cells.

### 3.8. Histological Examination

**Figure 9:**
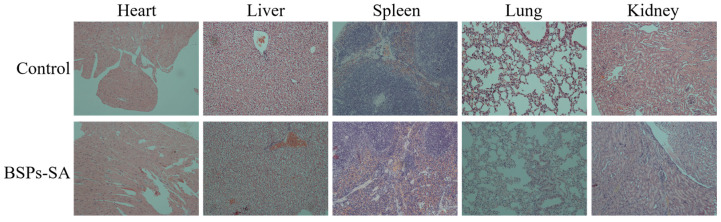
Histological images of heart, liver, lung, spleen, and kidney. The histological examination images are offered with a magnitude 200×.

Based on the above results, the BSPs-SA conjugate was selected for histological evaluation. Five major organs (the heart, liver, spleen, lung, and kidney) were assessed at a microscopic level for possible toxicity. No inflammation, necrosis, edema, or other pathological phenomena were observed in any of the organs at a dose of 200 mg/kg of BSPs-SA conjugate (Figure 9). All mice showed normal behavior, and there was no mortality associated with BSPs-SA copolymer treatment. The results demonstrated that the BSPs-SA conjugate had good biosafety.

### 3.9. Fluorescence Spectroscopic Measurement

**Figure 10:**
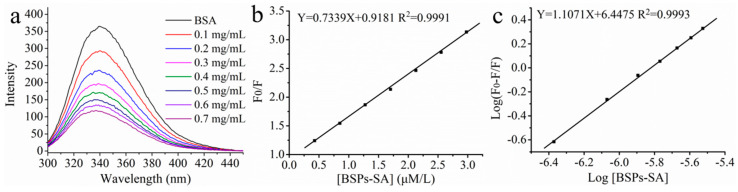
The fluorescence spectra (**a**) of bovine serum albumin (BSA) in a series of concentrations of BSPs-SA nanoparticles in deionized water (*T* = 298 K, λ ex = 293 nm). (**b**) The Stern–Volmer plots for the binding of BSPs-SA nanoparticles with bovine serum albumin. (**c**) Plots of log (*F*_0_-*F*/*F*) vs. log [BSPs-SA] at 298 K. (*n* = 3). The concentration of bovine serum albumin remained constant at 0.33 mg/mL. F_0_ and F are the fluorescence intensities at 340 nm in the absence and presence of BSPs-SA, respectively.

Fluorescence quenching technique is an effective method in exploring the binding ability, binding mechanism and conformational changes of bovine serum albumin with nanoparticles. The intrinsic fluorescence of bovine serum albumin is mainly due to the phenylalanine (Phe), tyrosine (Tyr), and tryptophan (Trp) residues [44]. The spectra of bovine serum albumin after incubation with BSPs-SA nanoparticles in a series of concentrations are shown in Figure 10a. Free bovine serum albumin exhibited a maximum emission at 340 nm, upon excitation at 293 nm. The emission fluorescence intensity distinctly weakened with the increase of BSPs-SA nanoparticles concentrations, which may be attributed to the formation of the non-fluorescent complex [32]. Moreover, a slight blue shift (2 nm) was observed in the maximum emission wavelength of bovine serum albumin, possibly due to the hydrophobic interaction between Trp residues of bovine serum albumin and hydrophobic segments (–CH_2_–, –CH_3_–, –C=O–) of BSPs-SA nanoparticles, causing a decrease in polarity or an increase in the hydrophobicity of the surrounding environment [45].

Fluorescence quenching may be due to two mechanisms, static quenching or dynamic quenching [28,29]. To clarify the quenching mechanism, static and dynamic processes were analyzed by the Stern–Volmer Equation (5).

(5) $$F_{0}/F=1+K\mathrm{sv}[Q]=1+kq\tau_{0}[Q]$$

where *F*_0_ and *F* represent the fluorescence intensities of bovine serum albumin in the absence and presence of quenchers (BSPs-SA nanoparticles), respectively. The *kq* and *K*sv are the quenching rate constant and the Stern–Volmer quenching rate constant of biomolecules, respectively. τ_0_ represents the average lifetime of bovine serum albumin without quencher, which is approximately 10^−8^ s. [Q] represents the concentration of quencher. The *K*sv value was 7.33 × 10^5^ L mol^−1^ and could be obtained from the slope of *F*_0_/*F* *vs.* [*Q*] in Figure 10b. In the present study, the value of *kq* (7.33 × 10^13^ L mol^−1^ s^−1^) was distinctly higher in comparison to the maximum diffusion rate constant (2.0 × 10^10^ L mol^−1^ s^−1^), which suggested that the quenching mechanism of bovine serum albumin with BSPs-SA nanoparticles was mainly governed by static quenching [30]. The binding constant (*K*_b_) and the number of the binding sites (*n*) could be calculated by Equation (6) to evaluate the possibility of BSPs-SA nanoparticles binding to bovine serum albumin.

(6) $$\log(F_{0}-F/F)=\log K_{b}+n\log[Q]$$

where *K*b is the binding constant and *n* represents the number of binding sites, respectively.

The values of *n* (1.107) and *K*_b_ (2.80 × 10^6^ L mol^−1^) are calculated from the slope and intercept of the plots of log[*F*_0_-*F*/*F*] vs. log[Q], respectively (Figure 10c). The value of *n* close to 1 indicates that there is one binding site between the bovine serum albumin and nanoparticles [46]. Generally, ligands can bind reversibly to bovine serum albumin and exhibit average binding affinities in the range of (1–15) × 10^4^ L mol^−1^. Hence, the *K*_b_ value of the bovine serum albumin BSA/BSPs-SA complex illustrated that there was a strong binding affinity, but the binding could not alter the conformation of bovine serum albumin.

### 3.10. UV Spectroscopic Measurement

**Figure 11:**
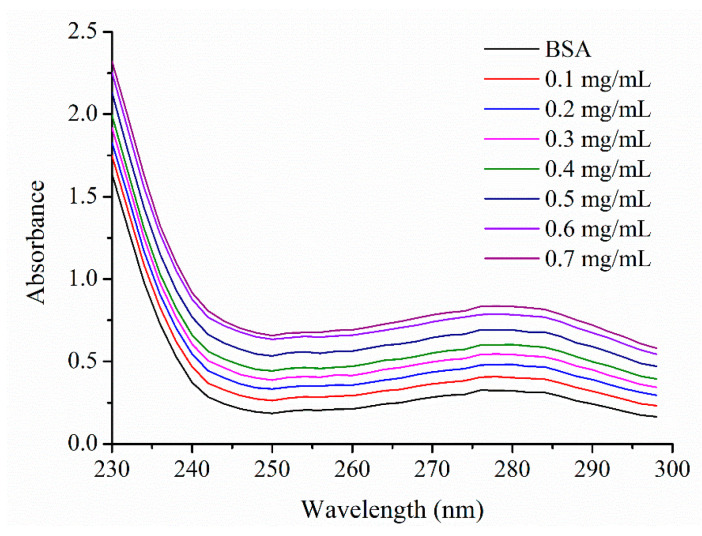
UV spectra of bovine serum albumin (BSA, 0.33 mg/ mL) in the presence of BSPs-SA nanoparticles (concentrations of BSPs-SA nanoparticles from top to bottom: 0, 0.1, 0.2, 0.3, 0.4, 0.5, 0.6, and 0.7 mg/mL).

To achieve more information about the affinity intensity of bovine serum albumin and BSPs-SA nanoparticles, UV spectroscopy was employed to evaluate the structural changes of bovine serum albumin subjected to BSPs-SA nanoparticles. Both bovine serum albumin and the BSA/BSPs-SA complex exhibited UV absorbance peaks at 278 nm as shown in Figure 11. The UV absorbance of bovine serum albumin displayed a weak absorption band at 278 nm owing to the weak absorption of three amino acids (Trp, Tyr, and Phe), which are located in the hydrophobic core of bovine serum albumin [47]. Compared with the spectra of free bovine serum albumin, the absorbance of the BSA/BSPs-SA complex at 278 nm gradually increased. The UV absorbance of the BSA/BSPs-SA complex at 278 nm was associated with the hydrophobic groups of bovine serum albumin and the –C=O– groups of BSPs-SA nanoparticles. BSPs-SA nanoparticles could absorb UV light. As shown in Figure 1, there were many hydrophilic moieties containing hydroxyl groups (–OH) and hydrophobic groups, such as methyl (–CH_3_) groups, methylene (–CH_2_–) groups, carboxide (–C=O–) groups, and glucosidic rings in the BSPs-SA conjugates. The amphiphilic BSPs-SA conjugates could spontaneously form a hydrophobic domain. Hydrophobic Trp and Try residues of bovine serum albumin are prone to combination with the hydrophobic domain of nanoparticles [48]. The hydrophobic interaction between BSPs-SA nanoparticles and bovine serum albumin induced the polarity change of the microenvironment around Trp and Try residues of the bovine serum albumin. It was seen that the absorbance of BSPs-SA nanoparticles or the BSA/BSPs-SA complex presented a concentration-dependent increase pattern in the range of concentration of 0.1–0.7 mg/mL (supporting information in Tables S1 and S2). It was noted that absorbance values of the BSA/BSPs-SA complex were obviously higher compared with that of BSPs-SA nanoparticles or pure bovine serum albumin bearing an absorbance value of 0.3248 at the concentration of 0.33 mg/mL, but not the sum of the individual absorbance value of pure bovine serum albumin solution and BSPs-SA nanoparticles. The results suggested that there was an interaction between BSPs-SA nanoparticles and bovine serum albumin. The interaction could affect microenvironment around the Trp and Try residues of the bovine serum albumin. Additionally, when the concentration of BSPs-SA nanoparticles exceeded 0.3 mg/mL (supplying the absorbance values of >0.1), this could influence the absorbance values of the BSA/BSPs-SA complex. Nevertheless, BSPs-SA nanoparticles at a concentration below 0.3 mg/mL had no impacts on the absorbance values of the BSA/BSPs-SA complex, while the absorbance of the BSA/BSPs-SA complex still displayed a concentration-related increase tendency higher compared with that of pure bovine serum albumin. Accordingly, the UV spectra of the BSA/BSPs-SA complex were not the result of UV-light absorption by BSPs-SA nanoparticles but of the binding ability between BSPs-SA nanoparticles and bovine serum albumin, which enabled the verification of the interaction between bovine serum albumin and BSPs-SA nanoparticles. We also found that the maximum UV absorption peak of the BSA/BSPs-SA complex presented no shift with the increase of BSPs-SA nanoparticles concentrations. The results implied that the microenvironment of bovine serum albumin was slightly changed due to intermolecular interactions, for instance, Van der Waal forces, hydrogen bonding, and electrostatic interactions. The results were the same as those reported in prior works [49,50].

### 3.11. Pharmacokinetics and Tissue Distribution

**Figure 12:**
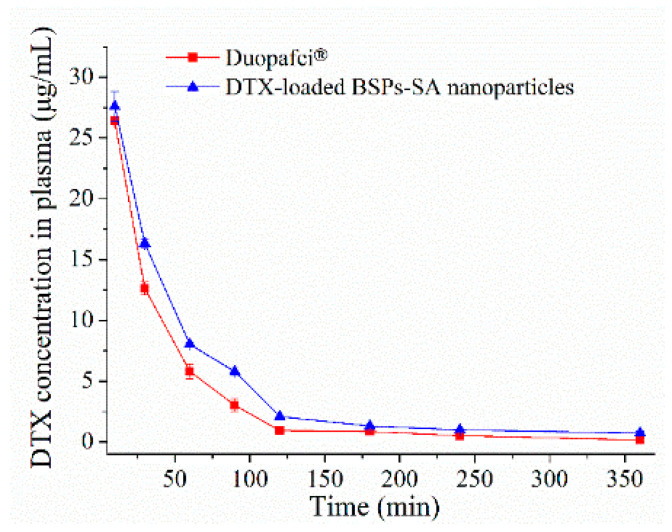
The mean plasma concentration–time curves of docetaxel (DTX) in 4T1 tumor-bearing BALB/c mice after a single tail vein injection of 25 mg/kg Duopafei^®^ and 25 mg/kg DTX-loaded BSPs-SA nanoparticles. Data are presented as mean ± SD (*n* = 5).

In our previous study [20], we evaluated the in vivo bioavailability of DTX-loaded BSPs-SA nanoparticles and Duopafei^®^ in healthy male Wistar rats. The results demonstrated that the absolute bioavailability of DTX-loaded BSPs-SA nanoparticles was 1.39-fold higher than that of Duopafei^®^. However, the physical function, species, and gender of 4T1 tumor-bearing BALB/c mice were different from the healthy rats. Hence, BALB/c female mice bearing 4T1 cancers were chosen to evaluate the pharmacokinetics and distribution of DTX-loaded BSPs-SA nanoparticles and Duopafei^®^. The detection limit (S/N = 3) and quantitation limit (S/N = 10) for docetaxel was 0.1 μg/mL and 0.2 μg/mL, respectively. The regression equation was as follows: *Y* = 22889*X* − 239.31. The linear range for docetaxel levels was in the range of 0.1–50 μg/mL (*R*^2^ = 0.9998). The concentration of docetaxel in mice subjected to Duopafei^®^ and DTX-loaded BSPs-SA copolymer nanoparticles at 6 h after vein injection were 0.16 ± 0.03 μg/mL and 0.81 ± 0.17 μg/mL, respectively. Therefore, the pharmacokinetic study is not conducted over a long period. The plasma concentration–time profiles of Duopafei^®^ and DTX-loaded BSPs-SA nanoparticles are presented in Figure 12 and the major pharmacokinetic parameters are shown in Table 1. This data indicated that the pharmacokinetics of DTX-loaded BSPs-SA nanoparticles were different from those of Duopafei^®^. The results were similar to those in our previous reports [20]. The area under the concentration–time curve (AUC_0–∞_) of DTX-loaded BSPs-SA nanoparticles was 30.72 ± 1.22 h mg/L, which was 1.42-fold higher than that of Duopafei^®^ (AUC_0-∞_ of 21.60 ± 1.07 h mg/L). The biological half-time of DTX-loaded BSPs-SA nanoparticles (t_1/2_, 1.13 ± 0.06 h) was approximately 1.36-fold longer than that of Duopafei^®^ (*t*_1/2_, 0.83 ± 0.05 h). One possible reason is ascribed to the particle size of 130 nm which avoided being recognized by macrophages and minimized the clearance rate [9,51]. Another probable reason is due to the fact that lipophilic docetaxel is incorporated into the hydrophobic domain of BSPs-SA supplying the sustained release and avoids being metabolized by enzymes in the liver [7,8,52]. The results of docetaxel release showed a significantly sustained release behavior for a relatively longer time after undergoing a fast release at the beginning. Accordingly, although the release rate of Duopafei was found to be faster than that of DTX-loaded BSPs-SA nanoparticles in the same aqueous media, DTX-loaded BSPs-SA nanoparticles presented a more superior and potent antitumor effect in comparison to Duopafei^®^. The mean residence time (MRT_0–∞ h_, 1.37 ± 0.12 h) of the DTX-loaded BSPs-SA nanoparticles was distinctly longer (1.69-fold) than that observed for Duopafei^®^ (MRT_0–∞ h_, 0.81 ± 0.06 h), which might be attributed to the sustained release of DTX-loaded BSPs-SA nanoparticles. The clearance rate (CL) of Duopafei^®^ (1.16 ± 0.06 L/h/kg) was 1.41-fold more rapid than that of DTX-loaded BSPs-SA nanoparticles (0.82 ± 0.03 L/h/kg). BSPs-SA nanoparticles delayed the elimination of docetaxel, which may be due to the fact that lipophilic docetaxel was incorporated into the hydrophobic core of BSPs-SA, which protected docetaxel from elimination by enzymes in the liver [8,52]. The special core–shell structure delayed the degradation and slowed down the release of docetaxel compared to Duopafei^®^.

**Figure 13:**
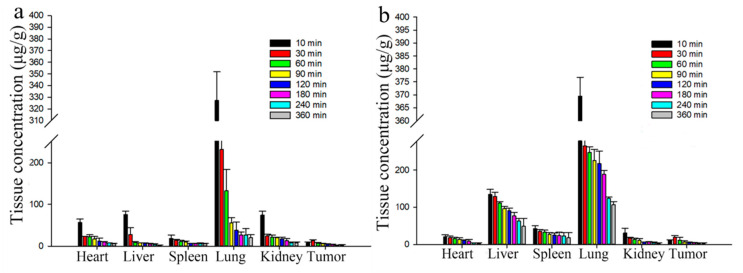
Concentration of DTX in different tissues at different time following via the tail vein dose (25 mg/kg) of Duopafei^®^ (**a**) and DTX-loaded BSPs-SA nanoparticles (**b**). Data are shown as mean ± SD. (*n* = 5).

**Figure 14:**
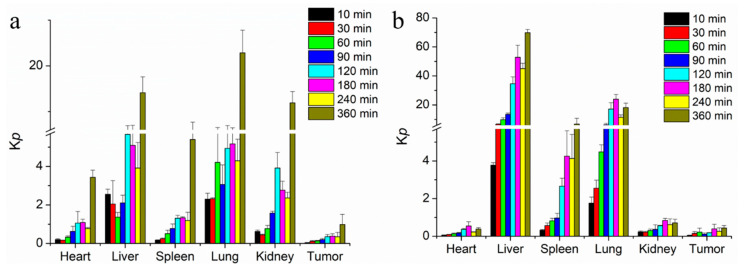
Ratio of tissue to plasma concentrations (*K*p) at different times following via the tail vein dose (25 mg/kg) of Duopafei^®^ (**a**) and DTX-loaded BSPs-SA nanoparticles (**b**). Data are shown as mean ± SD. (*n* = 5).

The docetaxel levels and *K*p values of Duopafei^®^ and DTX-loaded BSPs-SA nanoparticles in the heart, liver, spleen, lung, kidney, and tumor are shown in Figure 13 and Figure 14, respectively. For both Duopafei^®^ and DTX-loaded BSPs-SA nanoparticles, docetaxel concentrations in the lung were significantly higher than those in the heart, liver, spleen, and kidney, which may be ascribed to docetaxel sensitivity of lung cells [53]. In addition, docetaxel levels in the liver and spleen were greater than that of Duopafei^®^ in the corresponding organs, which demonstrated that the tissue distribution of docetaxel could be altered through being incorporated into BSPs-SA nanoparticles. In the Duopafei^®^ group, the docetaxel levels in the lung reached its maximum concentration of 327.21 ± 24.6 μg/g at 10 min, whereas the maximum concentration of the DTX-loaded BSPs-SA nanoparticles group was 369.48 ± 79.95 μg/g at 10 min. The *K*p values of Duopafei^®^ group and DTX-loaded BSPs-SA nanoparticles group in the lung were 2.30 ± 0.31 and 1.76 ± 0.32, at 10 min after vein injection, respectively. Docetaxel levels in the heart (5.98 ± 1.16 μg/g, *K*p = 3.43 ± 0.37) and kidney (7.25 ± 2.21 μg/g, *K*p = 11.90 ± 2.47) were higher in the Duopafei^®^ group than in the DTX-loaded BSPs-SA nanoparticles group (3.95 ± 0.26 μg/g, *K*p = 0.38 ± 0.07 in heart and 3.07 ± 0.97 μg/g, *K*p = 0.71 ± 0.20 in kidney) 6 h after tail vein injection. These results suggested that loading docetaxel into nanoparticles might reduce toxicity to the heart and kidney. It was noted that the docetaxel levels in the spleen and liver of the mice treated with DTX-loaded BSPs-SA nanoparticles group were 18.30 ± 13.79 μg/g and 49.10 ± 21.50 μg/g, respectively, 6 h after injection, which were dramatically higher than that of Duopafei^®^ group (5.31 ± 0.96 μg/g and 2.29 ± 0.63 μg/g, respectively). Similarly, *K*p values of the spleen (6.79 ± 3.93) and liver (69.83 ± 2.19) were higher in the DTX-loaded BSPs-SA nanoparticles group compared to the Duopafei^®^ group (5.39 ± 2.28 in spleen and 14.12 ± 3.48 in liver) 6 h via tail vein injection. This phenomenon might be explained by the fact that the liver and spleen are rich in macrophages [54]. It has been reported that macrophages are important mediators of nanoparticles uptake and drug release [55]. In addition, DTX-loaded BSPs-SA nanoparticles may be recognized as a foreign substance and could be taken up by the mononuclear phagocyte system [17].

It was seen that DTX-loaded BSPs-SA nanoparticles could enhance the accumulation amount of docetaxel in tumor sites. For the Duopafei^®^ group and the group treated with DTX-loaded BSPs-SA nanoparticles, the maximum concentrations of docetaxel in tumors were 13.09 ± 2.47 μg/g and 19.32 ± 4.83 μg/g at 30 min post injection, respectively. The *K*p values of DTX-loaded BSPs-SA nanoparticles group and Duopafei^®^ group were 0.15 ± 0.09 and 0.11 ± 0.04 at 30 min after vein injection, respectively. Additionally, we observed that the mice treated with DTX-loaded BSPs-SA nanoparticles and Duopafei^®^ displayed an obvious reduction in tumor weight and volume in comparison to those in the model control group (* *p* < 0.05). One reason for the increased accumulation of docetaxel in the tumors of nanoparticle-treated mice may be that the nanoparticles are taken up into cancer cells by endocytosis, improving docetaxel accumulation [39,56]. Again, the prolonged release of docetaxel from DTX-loaded BSPs-SA nanoparticles might be another reason [38,39].

## 4. Conclusions

In the present study, we found that the DS value of stearic acid in BSPs-SA conjugates distinctly affected their particle size, zeta potential, critical aggregation concentration, encapsulation efficiency, and drug-loading capacity as well as in vitro docetaxel release behavior. The results of X-ray diffraction and differential scanning calorimetry indicated that docetaxel was sequestered into the hydrophobic core of BSPs-SA nanoparticles and dispersed in small molecules or in an amorphous state. The release percentage corresponded to the DS value, with a slight decrease with an increase in the DS value. The docetaxel release from DTX-loaded BSPs-SA nanoparticles displayed an initial faster release and then a slower constant release, which is advantageous for clinical applications. Both Duopafei^®^ and DTX-loaded BSPs-SA nanoparticles enabled to a drop-down in the viability of 4T1 tumor cells in a dose-dependent pattern. Notably, the inhibitory effect of DTX-loaded BSPs-SA nanoparticles against 4T1 tumor cells was obviously superior to that of Duopafei^®^. The quenching mechanism of the bovine serum albumin and BSPs-SA nanoparticles was static. The interaction between bovine serum albumin and BSPs-SA nanoparticles exhibited a strong binding affinity. The biodistribution of docetaxel could be altered after being enclosed into BSPs-SA nanoparticles. Docetaxel levels in liver, spleen, and the tumor were higher, whereas they were lower in the heart and kidney, which is advantageous for lessening the toxicity for heart and kidney as compared with Duopafei^®^. Particularly, the enrichment of docetaxel in the tumor was beneficial to ensure the therapeutic effect. In conclusion, BSPs-SA nanoparticles might be a prospective drug delivery system, particularly for hydrophobic anticancer drugs.

## Figures and Tables

**Table 1:** Plasma pharmacokinetic parameters of DTX-loaded BSPs-SA nanoparticles and Duopafei^®^ with a dose of 25 mg/kg docetaxel after tail vein injection. Data are presented mean ± SD (*n* = 5).

Parameters	Duopafei^®^	DTX-Loaded BSPs-SA Nanoparticles
*t*_1/2_ (h)	0.83 ± 0.05	1.13 ± 0.06*
CL (L/h/kg)	1.16 ± 0.06	0.82 ± 0.03*
MRT_0–∞_ (h)	0.81 ± 0.06	1.37 ± 0.12*
AUC_0–6 h_ (h mg/L)	21.41 ± 1.04	29.38 ± 0.91*
AUC_0–∞_ (h mg/L)	21.60 ± 1.07	30.72 ± 1.22*

* Significantly different from Duopafei^®^ (*p < 0.05) by Student’s *t*-test. MRT: mean residence time; AUC: area under the concentration–time curve; CL: clearance rate; *t*_1/2_: biological half-time.

## Supplementary Materials

The following are available online at https://www.mdpi.com/1999-4923/11/1/43/s1, Table S1: Absorbance values of blank BSPs-SA nanoparticles at the concentration of 0.1–0.7 mg/mL; Table S2: Absorbance values of BSA/BSPs-SA complex at the concentration of 0.1–0.7 mg/mL.
