# Correction: Zhang et al. Interactions of Self-Assembled *Bletilla Striata* Polysaccharide Nanoparticles with Bovine Serum Albumin and Biodistribution of Its Docetaxel-Loaded Nanoparticles. *Pharmaceutics* 2019, *11*, 43

**DOI:** 10.3390/pharmaceutics17010015

**Published:** 2024-12-25

**Authors:** Guangyuan Zhang, Jin Qiao, Xin Liu, Yuran Liu, Ji Wu, Long Huang, Danyang Ji, Qingxiang Guan

**Affiliations:** Department of Pharmaceutics, School of Pharmacy, Jilin University, Changchun 130012, China; zhanggy17@mails.jlu.edu.cn (G.Z.); qiaojin17@mails.jlu.edu.cn (J.Q.); liux@jlu.edu.cn (X.L.); liuyr18@mails.jlu.edu.cn (Y.L.); wuji18@mails.jlu.edu.cn (J.W.); huanglong18@mails.jlu.edu.cn (L.H.); jidy16@mails.jlu.edu.cn (D.J.)

## Error in Figure

In the original publication [1], there was a mistake in Figure 9 as published. Due to the authors’ mistake, the staining images in the Control group and in the BSPs-SA group were placed incorrectly. The corrected Figure 9 appears below. The authors state that these corrections do not significantly impact the overall findings and conclusions of the paper. This correction was approved by the Academic Editor. The original publication has also been updated.


